# Yanghe Pingchuan Granules Alleviate Airway Inflammation in Bronchial Asthma and Inhibit Pyroptosis by Blocking the TLR4/NF-*κ*B/NRLP3 Signaling Pathway

**DOI:** 10.1155/2022/6561048

**Published:** 2022-08-31

**Authors:** Lingyu Pan, Yan Chen, Yeke Jiang, Yehong Sun, Yanquan Han, Yongzhong Wang

**Affiliations:** ^1^The First Affiliated Hospital of Anhui University of Chinese Medicine, Hefei, 230031 Anhui, China; ^2^Anhui University of Chinese Medicine, Hefei, 230012 Anhui, China

## Abstract

Bronchial asthma (BA) is a chronic inflammatory disease of the airway. Previous research has shown that Yanghe Pingchuan granules (YPGs) exert a precise therapeutic effect on BA. In our previous work, we showed that YPGs improved inflammation of the airways in rat models of BA. Other studies have shown that the pathogenesis of BA is closely related to pyroptosis and that the TOLL-like receptor pathway plays a key role in the mediation of pyroptosis. Therefore, in the present study, we established a rat model of BA by applying the concept of pyroptosis and used the TLR4/NF-*κ*B/NRLP3 signaling pathway as the target and YPGs as the treatment method. We evaluated the effects of YPGs on airway inflammation and pyroptosis in the model rats by HE staining, Masson's staining, AP-PAS staining, western blotting, and real-time quantitative PCR. The results showed that Yanghe Pingchuan granules could significantly improve the inflammatory response of bronchial tissue in BA rats, reduce the content of inflammatory factors IL-1*β* and IL-18, and inhibit the expression of pyroptosis factor. Meanwhile, YPG can block the TLR4/NF-*κ*B signaling pathway. These findings suggest that YPG may be an effective drug for the treatment of BA by blocking the TLR4/NF-*κ*B signaling pathway and inhibiting pyroptosis.

## 1. Introduction

Bronchial asthma (BA) is a major disease that poses significant threat to human health. The incidence of BA in children is relatively high and is showing an increasing trend over time, thus creating a serious burden on families and society [1]. However, existing drugs and treatments are not able to effectively prevent the occurrence of asthma. BA is a chronic form of airway inflammation that is created by the joint action of multiple cells and cellular components and also represents an immune disorder with a highly complex pathological mechanism [2, 3]. The main pathological manifestations of BA are inflammation, hyperresponsiveness, and remodeling in the airway [4]. Controlling inflammation has become one of the key methods for treating BA.

Pyroptosis is a newly discovered form of proinflammatory programmed cell death and is associated with a range of inflammatory factors, including interleukin-1*β* (IL-1*β*) and interleukin-18 (IL-18). The excessive maturation and release of these factors can induce an inflammatory cascade reaction [5]. The occurrence of pyroptosis is mediated by inflammasomes (mainly the NLRP3 inflammasome) and caspases (caspase-1/4/5/11), along with the release of a large number of inflammatory factors [6]. The formation of inflammasomes is a typical characteristic of cell pyroptosis in which Toll-like receptors (TLR) on the cell membrane recognize extracellular risk-related molecular patterns or pathogen-related molecular patterns. For example, TLR4 recognizes the activation of the NF-*κ*B signaling pathway by bacterial lipopolysaccharide (LPS), thus inducing the expression of NOD-like receptor proteins (such as NLRP3); the classic inflammasome process underlying the activation of the inflammasome involves pro-IL-1*β* and pro-IL-18 [7, 8]. The activation of inflammasomes is one of the main causes of airway inflammation in BA. In recent years, studies have confirmed that the substrate for inflammatory caspase is gasdermin D (GSDMD), a substance that causes pyroptosis by creating small holes in the cell membrane following lysis [9]. The occurrence of pyroptosis is often accompanied by the increase in characteristic inflammatory factors, such as IL-1*β* and IL-18. Research has shown that IL-1*β* can cause tissue inflammation, vasodilation, and immune cell extravasation; however, IL-1*β* is also adaptive and plays a role in the immune response. IL-18 has been shown to promote Th1 cells, NK cells, and cytotoxic T cells to produce interferon-*γ* (IFN-*γ*); it can also promote the development and maturation of Th2 cells and enhance local inflammatory responses [10, 11]. Therefore, pyroptosis is closely related to the occurrence of allergic diseases such as BA, allergic rhinitis, atopic dermatitis, and urticaria.

Toll-like receptor TLR4 is closely related to the occurrence of pyroptosis [12]. TLR4 mediates the downstream translocation of nuclear transcription factor *κ*B (NF-*κ*B) into the nucleus by binding to LPS, thereby activating NF-*κ*B, upregulating the expression levels of NLRP3, IL-1*β*, and IL-18, regulating the inflammasome, and causing pyroptosis [13, 14]. Although several factors can activate the NLRP3 inflammasome, the classic and most established signaling system is the TLR4/NF-*κ*B/NRLP3 pathway [15]. Activated TLR4 combines with the primary response protein of bone marrow differentiation (MyD88) to form a signal complex that activates NF-*κ*B through a complex cascade reaction, thereby initiating and regulating numerous inflammatory mediators, adhesion molecules, and enzymes related to the inflammatory immune response [16]. Research has shown that the TLR4/NF-*κ*B/NRLP3 signaling pathway plays a key role in the occurrence and development of many allergic inflammatory diseases.

Yanghe Pingchuan granules (YPGs) are a medication prepared at the First Affiliated Hospital, Anhui University of Chinese Medicine. YPGs are composed of Ephedra sinica Stapf., Rehmannia glutinosa (Gaertn.) DC, Inula japonica Thunb., Morinda officinalis F.C.How, Schisandra chinensis (Turcz.) Baill., Sinapis alba L., Draba nemorosa L., Angelica sinensis (Oliv.) Diels, and Platycodon grandiflorus (Jacq.) A.DC. YPGs are known to invigorate the kidney, activate the blood, resolve phlegm, and relieve cough and asthma [17]. YPGs have also been used in the clinical treatment of BA for many years with clear curative effects [18, 19].

In our previous research, we confirmed that YPGs exerted a definite therapeutic effect on airway remodeling and airway inflammation in a rat model of BA and described quality control experiments for these granules [20]. Many studies have shown that pyroptosis may be one of the key causes of BA [21, 22]. The Toll-like receptor signaling pathway is a key pathway for the occurrence of pyroptosis which can lead to high expression levels of interleukin-like inflammatory factors. Therefore, we hypothesized that the abnormal activation of the TLR4/NF-*κ*B/NRLP3 signaling pathway will promote pyroptosis in the smooth muscle cells of the airway, mediate caspase-1-dependent pyroptosis in the smooth muscle cells of the airway, cause a strong inflammatory response, and induce BA. Based on the theory of pyroptosis, our present study applied a series of detection methods to investigate the mechanisms underlying the effects of YPGs in the treatment of BA by regulating the TLR4/NF-*κ*B/NRLP3 signaling pathway.

## 2. Materials and Methods

### 2.1. Establishment of a BA Model

**Figure 1:**
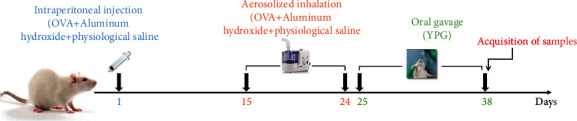
Rat model of bronchial asthma and treatment with YPG.

Specific pathogen-free (SPF) male Sprague-Dawley (SD) rats, weighing 220 ± 30 g, were provided by Anhui Medical University (production license: SCXK (Anhui) 2017-001). The rats were randomly divided into a normal group (N), an asthma model group (M), a YPG group (YPG), an NF-*κ*B blocker (CID2858522) group, and a positive control (Guilong Kechuanning capsule) group (GK); each group featured 10 rats.  Figure 1 shows the establishment of the OVA-induced BA rat model and treatment procedure. On the first day of the experiment, the rats in each group (except for the normal group) were injected intraperitoneally with 10% OVA+10% aluminum hydroxide+physiological saline (1 mL) to induce sensitivity. On the 15^th^ day of the experiment, the sensitized rats were placed in a nebulizer box and exposed to the aerosolized inhalation of 1% egg albumin+physiological saline (at a constant pressure of 400 mmHg) for 15 min, 2 d/1 time, for 10 d, to establish a rat model of asthma.

On the 28^th^ day after modeling, rats in the YPG groups were administered with YPGs (14.76 g/kg, once a day) by oral gavage. The NF-*κ*B blocker group was administered with a pathway blocker (CID2858522). The positive control group received Guilong Kechuanning capsules (1.0 g/kg, once a day) by oral gavage. The normal group and model group were given the same amount of normal saline once a day for 14 consecutive days. At the end of the experiment, all rats were fasted for 8 hours, weighed, and anesthetized by the intraperitoneal injection of 2% pentobarbital sodium (40 mg/kg). Then, the bronchial tissues were collected in freezing tubes and stored at −80°C to await analysis.

All animal experiments were approved by the Animal Experiment Ethics Committee of Anhui University of Chinese Medicine (license number LLSC20160336).

### 2.2. Chemicals and Instruments

YPGs were prepared by the First Affiliated Hospital of Anhui University of Chinese Medicine (batch number 20201011). Guilong Kechuanning capsules were provided by Guilong Pharmaceuticals (Anhui Co. Ltd., Maanshan). NF-*κ*B and OVA were provided by Sigma-Aldrich Co. (MO, USA). The RNA extraction kit and fluorescence quantitative PCR kit were provided by Sangong Bioengineering (Shanghai Co., Ltd.). We also used a real-time PCR instrument (Roche, Switzerland, 480II) and an ultraviolet spectrophotometer (Nanodrop2000; Thermo, USA).

### 2.3. Bronchoalveolar Lavage Fluid (BALF) Collection and Differential Cell Counts

After the rat was intubated by the trachea, 2 mL of ice-cold sterile PBS buffer was slowly injected, and lavage was performed three times, and the obtained solution was the bronchoalveolar lavage fluid. The bronchoalveolar lavage fluid was centrifuged at low temperature (4°C, 3000 rpm, 5 minutes), and the supernatant was aliquoted and stored at -80°C. The cell pellets harvested from BALF were resuspended in 200 *μ*L of PBS. A hemocytometer and the Wright-Giemsa staining were used to assess the total number of white blood cells and the proportion of inflammatory cells (eosinophils, macrophages, neutrophils, and lymphocytes), respectively; 200 cells/slide were counted.

### 2.4. Hematoxylin and Eosin (H&E) Staining

The right lungs were fixed, embedded in paraffin, and sectioned. Changes in the structure of the airway wall, along with pathological alterations of the bronchi and smooth muscles, were observed by conventional H&E staining and analyzed using the ImageJ software.

### 2.5. Masson's Staining

Tissues were fixed in 4% paraformaldehyde at 55°C for 1 h. The extent of bronchial fibrosis was then determined by Masson's trichrome (1%) staining for 5-10 min at 55°C. Then, we evaluated the extent of fibrosis in the lungs and bronchial tissue with an optical microscope and performed pathological grading.

### 2.6. Alcian Blue-Periodic Acid Schiff (AB-PAS) Staining

Sections were first deparaffinized in xylene, stained with Alcian blue solution (10-20 min), periodic acid oxidation (5 min), and Schiff's reagent (10-20 min), and then stained with Scott blue solution and ethanol. Following dehydration with a gradient series of alcohols, the sections were cleared with xylene and then observed by microscopy. This allowed us to investigate the secretion of acidic mucus in the lungs and bronchial tissue.

### 2.7. Enzyme-Linked Immunosorbent Assay

Bronchial alveolar lavage fluid was collected from each rat and centrifuged at 350 g for 5 min at 4°C. Supernatants were stored at −80°C to await cytokine analysis. ELISAs were used to quantify the levels of IL-1*β* and IL-18 and were carried out in accordance with the manufacturer's instructions. Optical density (OD) values were read at 450 nm, and the levels of IL-1*β* and IL-18 were determined by standard curves.

### 2.8. Real-Time Quantitative PCR

A TRIzol reagent was used to extract total RNA from each sample in accordance with the manufacturer's protocol. Then, 2 *μ*g of total RNA was used as a template for reverse transcription to synthesize cDNA. Quantitative fluorescence PCR, using an ABI7000 quantitative fluorescence monitor, was then used to determine the relative expression of *TLR4*, *NF-κB*, *NLRP3*, *GSDMD*, and *GSDME* mRNA. The primers are listed in Table 1.

### 2.9. Immunofluorescence

Samples were fixed with 4% paraformaldehyde for 20 min. Then, 0.5% Triton X-100 was diluted in PBS, added to 96-well plates, and incubated for 20 min. The sections were then rinsed three times with PBS and blocked for 30 min with 5% Bovine Serum Albumin (BSA) solution. Sections were then washed and incubated with primary antibodies (TLR4: 1 : 500; NF-*κ*B: 1 : 200; NLRP3: 1 : 50; GSDMD: 1 : 50; and GSDME: 1 : 400). Sections were then placed in a humid box and incubated overnight at 4°C in the dark. The following morning, sections were washed and incubated with corresponding secondary antibodies and incubated at room temperature for 50 min. 4′,6-diamidino-2-phenylindole (DAPI) was used to stain cell nuclei.

### 2.10. Western Blotting

Samples were washed three times with cold PBS for three times and cleaved with lysis buffer. Then, 100 *μ*L of each protein lysate was separated by sodium dodecyl sulfate polyacrylamide gel electrophoresis (SDS-PAGE). The separated proteins were then transferred onto polyvinylidene fluoride (PVDF) membranes and blocked in Tris-buffered saline Tween-20 (TBST) (washed with 1× TBST for 2 min and then sealed with 5% skimmed milk). Then, the PVDF membranes were incubated overnight at 4°C with the following primary antibodies: *β*-actin (1 : 5000, Abcam, Cambridge, UK), TLR4 (1 : 5000, Abcam, Cambridge, UK), NF-*κ*B ((1 : 6000, Proteintech, Chicago, USA), NLRP3 (1 : 2000, Proteintech, Chicago, USA), GSDMD (1 : 1000, Abcam, Cambridge, UK), and GSDME (1 : 1000, CST, Boston, USA). The following morning, the membranes were washed three times with TBST and incubated with a secondary anti-mouse or anti-rabbit antibody HRP (1 : 10000) for 2 h at room temperature. Antibody binding was then detected with an enhanced chemiluminescence kit. Western blotting data were quantified with the ImageJ software. Each protein was analyzed in triplicate.

## 3. Results

### 3.1. Effects of YPG on Inflammatory Cell Recruitment in BALF

**Figure 2:**
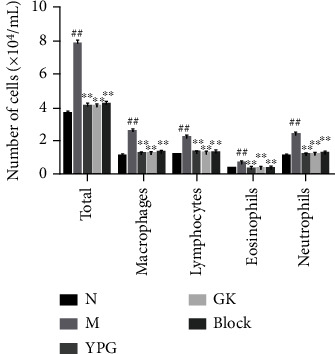
Effects of YPG on the number of inflammatory cells in BALF. Notes: ^##^*P* < 0.01 compared with the normal group; ^∗∗^*P* < 0.01 compared with the model group.

The results of inflammatory cell count are shown in Figure 2. Compared with the normal group, the total number of cells in the model group was significantly increased, and inflammatory cells (macrophages, lymphocytes, eosinophils, and neutrophils) were significantly increased (*P* < 0.01). After drug intervention in each group, compared with the model group, the total number of cells and the number of inflammatory cells in the YPG group, GK group, and block group were significantly decreased.

### 3.2. Pathological Changes

**Figure 3:**
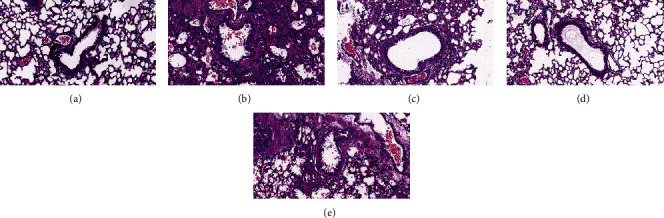
The effect of YPG on bronchial asthma in rats. Sections were stained with H&E. Magnification, ×400; scale bars, 50 *μ*m. Normal group (a), model group (b), YPG group (c), GK group (d), and pathway blocker group (e).

Figure 3 shows pathological changes of the bronchus in a rat model of BA before and after drug treatment. In the normal group, no inflammatory cell infiltration was seen in the bronchus; the bronchial wall was smooth, the cells were arranged regularly, and the thickness of the soft muscle layer was average. When compared with the normal group, the BA rats had a large extent of inflammatory cell infiltrations around the bronchial wall, along with a series of pathological changes, including microvascular leakage, the proliferation of goblet cells and airway epithelial cells, a thickening of the smooth muscle layer, and a thickening and elongation of the bronchial mucosa. After YPG treatment, inflammatory cell infiltration was significantly reduced, the thickness of the tube wall and smooth muscle layer was similar to that of the normal group, and the structure of the bronchial mucosa was much improved. The GKG group and the inhibitor group also showed clear therapeutic effects; the effects were more pronounced in the inhibitor group.

### 3.3. Bronchial Fibrosis and the Detection of Collagen Deposition

**Figure 4:**
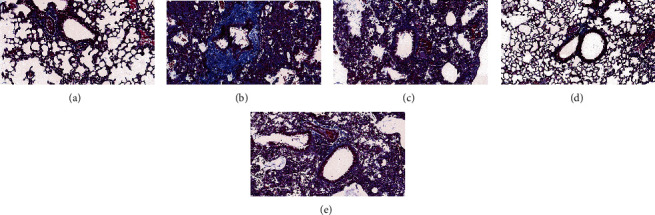
The effect of YPG on bronchial asthma in rats. Sections were stained with Masson's trichrome. Magnification, ×400; scale bars, 50 *μ*m. Normal group (a), model group (b), YPG group (c), GK group (d), and pathway blocker group (e).

Masson's trichrome staining showed that the structure of the lung tissue in the normal group was normal and that collagen fiber precipitation (blue) and muscle fibers (red) were rarely increased (Figure 4). Compared with the normal group, the bronchial wall and alveolar wall were obviously thickened and consolidated in the model group, with a significant increase in the areas stained blue and red. After drug treatment, the YPG group, the inhibitor group, and the GKG group all showed significant improvements in these symptoms; collagen fibers and muscle fibers were significantly reduced. The most significant improvement was evident in the inhibitor group.

### 3.4. The Detection of Bronchial Mucus Secretion

**Figure 5:**
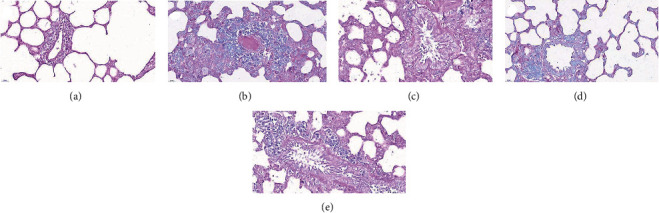
The effect of YPG on bronchial asthma in rats. Sections were stained with AB-PAS. Magnification, ×400; scale bars, 50 *μ*m. Normal group (a), model group (b), YPG group (c), GK group (d), and pathway blocker group (e).

AB-PAS staining was used to detect mucus secretion in the lungs and bronchi. Mucus is an important glycoprotein complex within the airways. The mucus material was stained blue in the presence of AB while neutral mucus material was stained purple by PAS. Goblet cells are an important source of mucus secretion. AB-PAS staining showed that there were fewer goblet cells in the normal group (Figure 5). Compared with the normal group, the number of goblet cells in the model group had increased significantly and a large amount of mucus was evident in the airway cavity. After drug treatment, each administration group showed significant reductions in the number of Goblet cells and mucus secretion.

### 3.5. The Detection of Pyroptosis

#### 3.5.1. The Expression of Inflammatory Factors

**Figure 6:**
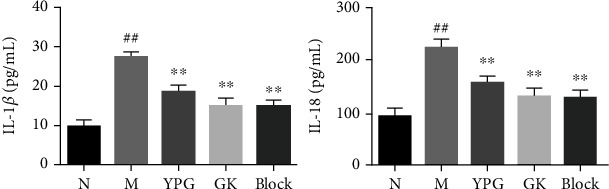
Effect of YPG on the expression of IL-1*β* and IL-18 in BA rats. Notes: levels of IL-1*β* and IL-18 were measured by ELISA. Data are expressed as means ± SD. *n* = 8. Notes: ^##^*P* < 0.01 compared with the normal group; ^∗∗^*P* < 0.01 compared with the model group.

ELISA was used to detect the expression levels of the inflammatory factors IL-1*β* and IL-18 in bronchial tissue. As shown in Figure 6, compared with the normal group, there was a significant increase in the expression levels of IL-1*β* and IL-18 in the model group. After drug interventions, the expression levels of IL-1*β* and IL-18 in the YPG, GLK, and CID2858522 groups were significantly decreased (*P* < 0.05). These results showed that YPG inhibited the expression of the inflammatory factor IL-1 in BA rats and that the mechanism involved may be related to the regulation of pyroptosis.

### 3.6. The Expression of the Pyroptosis Factor

**Figure 7:**
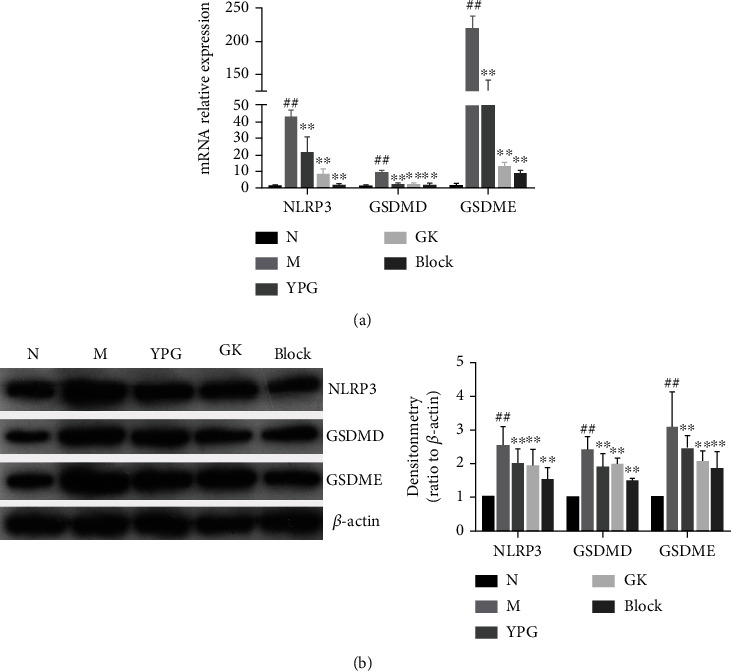
Effect of YPG on the expression of pyroptosis factor in BA rats. (a) mRNA levels of NLRP3, GSDMD, and GSDME were determined by real-time quantitative PCR. (b) The change in NLRP3, GSDMD, and GSDME protein expression was observed by western blotting. Notes: ^##^*P* < 0.01 compared with the normal group; ^∗∗^*P* < 0.01 compared with the model group.

Real-time quantitative PCR and western blotting were used to detect the mRNA and protein levels of NLRP3, GSDMD, and GSDME. As shown in Figure 7, compared with the normal group, the mRNA and protein levels of NLRP3, GSDMD, and GSDME in the model group were significantly increased. After drug interventions, the expression levels of NLRP3, GSDMD, and GSDME mRNA and protein were significantly decreased in each administration group. Generally, the effects observed in the pathway blocker group and the positive drug group were significantly better than those in the YPG group (*P* < 0.01).

### 3.7. The TLR4/NF-*κ*B Signaling Pathway

**Figure 8:**
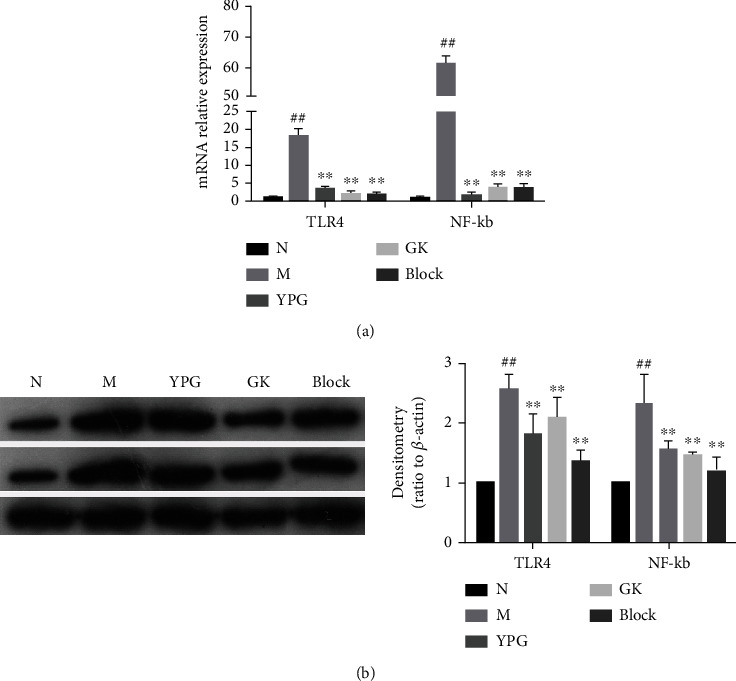
Effect of YPG on the expression of the pyroptosis factor in BA rats. (a) mRNA levels of TLR4 and NF-*κ*B were determined by real-time quantitative PCR. (b) The change in TLR4 and NF-*κ*B protein expression was observed by western blotting. Notes: ^##^*P* < 0.01 compared with the normal group; ^∗∗^*P* < 0.01 compared with the model group.

Real-time quantitative PCR and western blotting were used to detect the mRNA and protein levels of TLR4 and NF-*κ*B in the TLR4/NF-*κ*B signaling pathway. As shown in Figure 8, compared with the normal group, the mRNA and protein expression levels of TLR4 and NF-*κ*B in the model group were significantly increased. After drug interventions, the expression levels of TLR4 and NF-*κ*B mRNA were significantly decreased in each administration group. Generally, the effects observed in the blocker group and the positive drug group were significantly better than those in the YPG group (*P* < 0.01).

## 4. Discussion

BA is a chronic inflammatory disease of the airway involving a variety of inflammatory cells and inflammatory mediators [23]. These airway inflammatory cells include eosinophils, mast cells, lymphocytes, neutrophils, and macrophages. The pathological features of asthma in the airway are inflammation, hyperresponsiveness (airway high reactivity (AHR)), and remodeling. Airway inflammation is the basic pathological change associated with asthma and is predominantly manifested by eosinophil infiltration [24]. Eosinophilic granulocytes cause a series of inflammatory reactions by releasing granular proteins; this is an important cause of repeated asthma symptoms and the pathological basis for airway hyperresponsiveness. Therefore, reducing the release of inflammatory factors and controlling airway inflammation are effective methods for treating BA.

When considering the mechanisms involved in such inflammation, it is important to consider pyroptosis. This is a newly discovered form of programmed cell death that is caused by disturbances in the homeostasis of the extracellular or intracellular environment. The occurrence of pyroptosis depends on the activation of caspase-1 or caspase-4/5/11. When activated, caspase-1 or caspase-4/5/11 causes the further lysis of GSDMD; these punch holes in the cell membrane cause the cell to swell and the cell membrane to rupture. Finally, the cell contents are released outside into the extracellular environment, thus causing inflammation [25]. Therefore, pyroptosis is also referred to as cellular inflammatory necrosis [26]. Previous studies have shown that pyroptosis features classical and nonclassical pathways [9]. The classical pathway predominantly relies on caspase-1. When cells are stimulated by exogenous factors (bacteria, viruses) or endogenous factors (stress factors released by the body after damage), caspase-1 is activated and GSDMD undergoes lysis to cause the perforation of cell membranes; this results in changes in the osmotic pressure inside and outside the cells. A large amount of extracellular substances can then cause the cell to rupture and undergo necrosis; this process releases a large number of inflammatory factors and induces or aggravates the body's inflammatory response [27]. In addition, the activation of caspase-1 can also cleave the precursors of IL-18 and IL-1*β*, thus aggravating the inflammatory response. The nonclassical pathway of cell pyroptosis depends on caspase-4, caspase-5, and caspase-11; these enzymes can bind directly to bacterial lipopolysaccharide (LPS) to cause cellular necrosis and inflammation. These enzymes are also involved in the activation of caspase-1, thus releasing IL-1*β* and IL-18 to the extracellular environment, thus leading to the aggregation of inflammatory cells and an amplification in the inflammatory response.

The activation of caspase-1 in the classical pathway is directly associated with the inflammasome. According to different receptor proteins, inflammasomes can be divided into multiple subtypes, including NLRP3, NLRC4, AIM2, and NLRP1. Of these, NLRP3 has been confirmed to be related to many diseases and participates in various immune activities in the body which are mediated by microbial activity. Therefore, the detection of pyroptosis marker proteins such as GSDMD, GSDME, the NLRP3 inflammasome, and inflammatory factors IL-18 and IL-1*β* is essential if we are to confirm whether cells are undergoing pyroptosis.

In the previous experiments, we conducted a series of studies on YPG, including quality control and basic pharmacodynamics research. We determined that YPG exerts a specific therapeutic effect on BA and that the mechanism of action may be related to regulation of the PI3K/PKB signal pathway and an inhibition of the abnormal proliferation of smooth muscle in the airways. To explore whether YPG can treat BA by inhibiting pyroptosis, in the present study, we focused on the TLR4/NF-*κ*B/NRLP3 signaling pathway in order to validate our previous research. Experimental results showed that YPG significantly improved inflammation and fibrosis in the bronchus of BA rats and significantly reduced the number of pyroptosis bodies, thus indicating that YPG exerts a therapeutic effect on BA by inhibiting pyroptosis. Further studies showed that YPG significantly inhibited the expression of key genes and proteins, including TLR4, NF-*κ*B, NLRP3, GSDMD, and GSDME in the TLR4/NF-*κ*B/NRLP3 signaling pathway, thus indicating that YPG may block the TLR4/NF-*κ*B/NRLP3 pathway, thus inhibiting cellular pyroptosis.

## 5. Conclusion

Analysis showed that the pathogenesis of BA may be related to excessive activation of the TLR4/NF-*κ*B/NRLP3 signaling pathway, thus leading to the pyroptosis of smooth muscle cells in the airway and the induction of extensive inflammatory responses. YPG alleviates BA by blocking the TLR4 signaling pathway, thus inhibiting pyroptosis in the smooth muscle cells of the airway and reducing bronchial inflammation.

## Figures and Tables

**Table 1:** Real-time PCR primers.

Gene	Primers	Product length (bp)
TLR4	F: TTGCCTTCATTACAGGGACTT R: CAGAGCGGCTACTCAGAAACT	104

NF-*κ*B	F: ACGATCTGTTTCCCCTCATC R: TGCTTCTCTCCCCAGGAATA	127

GSDMD	F: CCCAGAAGCCCCACTATACC R: AAGGCAGTCCACTGGGATGA	124

GSDME	F: ATGGAGGACGTTTCTCACGG R: GCTCGAAGCCACCATGTTTC	166

NLRP3	F: ATGGTGTGCCAGGAAGACAG R: CCAGTTGGAGCTGGGTGTAG	150

## Data Availability

The data used to support the findings of this study are included within the article.
